# 

**Published:** 1855-06

**Authors:** 


### INDEX TO VOLUME ELEVENTH.

For original communications, look for the names of the authors as well as the titles of the subjects.

For selected and editorial articles, look for the titles of the subjects only.

For notices of books look for names of authors.

### ILLUSTRATIONS.

Portrait of Prof. Austin Flint, .... June No.
Case of Cyanosis, by Prof. White, .... July No.
Fracture of the Scapula, by Prof. Hamilton, .... January No.

### A.

PAGE.

Abortion with Placenta Prævia and Hydrops Amnii. By David W. Hershey, M. D., .... 141
Abortion following Chloroform, .... 558
Abortion: Spontaneous Inversion of the Uterus, .... 745
Abscess, Urinary, Dr. C. B. Hutchins on, .... 577
Address by Prof. C. B. Coventry, .... 178
" by Prof. J. C. Dalton, Jr., .... 446
" by Prof. J. P. White, .... 529
Air Passages, Dr. Wm. G. Meachem, on Local Treatment of, .... 69
American Medical Association,
Eighth Meeting of, .... 47
Reception of, .... 128
History of, .... 312
Card of, .... 444
Transactions of, .... 473
J. M. N. on next meeting of, .... 587
Ninth Meeting of, .... 704
Amputation at Knee Joint, .... 662
Anatomical Remembrancer, .... 447
Anecdotical, .... 254
Appointments at Buffalo Hospital, .... 319
Apoplexy, etc., .... 682
Army Meteorological Register, .... 603

PAGE.

Artificial Hand, .... 63
Ashwell on Women, .... 155

### B.

Barton on Yellow Fever, .... 313
Beasley's Prescription Book, .... 478
Beck, Death of T. Romeyn, .... 448, 491
Bedford on Diseases of Women, .... 233
Biography of Living Medical Men, .... 460
Blood, Independent Vitality of, by L. G. Robinson, M. D., .... 385
Blancard's Pills, .... 495, 618
Blessing, A Rare, .... 624
Bowman's Chemistry, .... 725
Bright's Disease, .... 663
Bronchial Injections, .... 697
Brain, Functions of, .... 247
Burwell, Dr. Geo., on Pneumonia, 87, 142, 275, 417, 705
Butler, Dr. Wm. C., on Stricture of Vagina, .... 411
" on Scarlatina, .... 534
Buffalo Med. College, .188, 252, 318, 638
Buffalo Medical Association, Proceedings of, .... 84, 142, 228, 270, 335, 416, 465, 519, 592, 641

**C.** PAGE.

Caldwell, Autobiography of Charles, 29
Catheterization of Air Passages, .... 181
Cancer, Epithelial, by William Van Pelt, M. D., .................... 227
Carpenter's Human Physiology, .... 361
Changes and Exchanges, .......... 124
Chemistry, Rand's Medical, ......... 33
Chandler, Dr. John L., on Medical Education, .................... 216
Cholera, Jamieson on, .............. 313
" Infantum, .................... 622
Clavicle, Prof. F. H. Hamilton, on Fractures of, .................... 437
Clergymen and Physicians, ......... 738
Climatology of the United States, ... 701
Compliment to Prof. Flint, .......... 46
Cogitations and Vaticinations, ...... 658
Correction, ........................ 255
" of an Error proved to be no Error, by Prof. S. D. Gross, ...... 631
Compression of Soft Parts during Labor, ........................ 553
Criminal Lunacy, .................. 563
Culinary, ........................ 169
Cyanosis, Case of, (Illustrated) by Prof. James P. White, ........... 65
Cyanosis, Case of, by E. Delany, M. D., ........................ 269
Czar's Case, ..................... 105

**D.**

Dalton, Jr., Address by Prof. John C., ........................... 446
Dayton, Dr. C. L., on Deaths in Buffalo, ..................610, 657, 727
Delany, Dr. Edmund, on Cyanosis, . 269
Deaths in Buffalo, Record of, by John Root, M. D., 36, 94, 162, 238, 316, 364, 412, 480, 544
" by C. L. Dayton, M. D., 610, 657, 727
Dislocations, Report on, by Prof. F. H. Hamilton, .................. 178
" of Femur into Ischiatic Notch reduced, by Manipulation, by Prof. Hamilton, ..................... 210
" of Femur, Reduction of, ...612, 613
Dickson's Elements of Medicine, ... 356
Dictionary of Medical Terms, ...... 362
Diseases, Specificness of, ........... 625

**E.** PAGE.

Epidemic Changes in Fevers, ...... 176
Erie Co. Med. Society, Transactions of, ...................... 120, 524
Extirpation of Uterus, ............. 40
Explanation, ................ 255, 689
Exopthalmia, by E. R. Maxson, M. D. 585
Eye, Clinical Lectures on, by Isodore Gluck, M. D., ............ 193, 322
" Mackenzie on, ................ 360
" Hyperæsthesia of, by Prof. S. B. Hunt, ........................ 645

**F.**

Fergusson, Wm., Portrait of, ....... 64
Fever, Causes of Yellow, ........... 313
" Epidemic Changes in, ....... 175
" Nourishment in, ............ 176
" Typhoid, by E. R. Maxson, M. D., ........................ 713
Female Physicians, ................ 732
Fracture of Scapula, by Prof. F. H. Hamilton, ..................... 449
" of Ribs, ....................... 621
Fistula in Ano, ................... 557
Flint, Prof. Austin, on Reduplication of Heart Sounds, ................ 1
" Compliment to, .............. 46
" Lectures on Diseases of the Skin, 129, 394
" Report on Pneumonia, ........ 288
" Letter on " ........ 513
" on Respiratory Organs, ........ 718
Flint, Jr., Austin, on Paronychia, ... 257

**G.**

Gay, Dr. C. C. F., Report on Pneumonia, ......................... 271
Gastrotomy, Case of, .............. 103
German Medical Society of Buffalo, . 63
Gluck, M. D., Isodore, on Opacities of the Cornea, ............ 193, 322
Glucogenetic Functions of the Lungs, 370
Gross, Prof. S. D., on Urinary Organs, 160
" Correction of an Error, by, ... 631
Green on Bronchial Injections, ...... 697

**H.**

Hamilton, Prof. F. H., Report on Dislocations, ...................... 178

PAGE.

Hamilton, Prof. F. H., on Dislocation of Femur, ........................ 210
" on Fracture of Clavicle, ......... 437
" " " of Scapula, ......... 449
" " Female Syringes, ........... 464
Happy New Year, ................... 498
Hall on Paralysis, .................. 690
Heart, Reduplication of Both Sounds of, by Prof. Austin Flint, ......... 1
" State of, in Fever, .............. 104
" Wounds of, ..................... 127
" Corson on Valvular Disease of, . 128
" and Aorta, Stokes on, ........... 360
Henderson on Homœopathy, ........ 34
Headland on Action of Medicines, .. 649
Hershey, Dr. D. W., on Placenta Prævia, ............................ 141
History of Amer'n Med. Association, 312
How to Nurse Sick Children, ...... 447
Holmes, Prof. O. W., Toast of, ...... 63
Homœopathy, ....................... 635
" Illustration of, ........ 688
" in Cholera, ........... 681
Hutchins, Dr. C. B., on Urinary Abscess, ........................... 577
Hyperæsthesia Oculi, Prof. S. B. Hunt, on, ......................... 645
Hypnotism in Hysterical Paralysis, . 670
Hunt, Prof. S. B., Sketch of Practical Meteorology, ................... 76
" on Meteorology of Buffalo, 35, 96, 164, 237, 315, 366, 411, 479, 543, 609, 656, 726.
" on Ocular Hyperæsthesia, ...... 645

I.

Iodine as an Antidote to Mercury, .. 736
Inhalation, ......................... 482
Intestinal Obstruction, ............. 546
Inversion of the Uterus, ............ 716
Inversion of the Uterus following Parturition, ......................... 745

J.

Jamieson on Cholera, ............... 313
Joke, A Very Good, ................. 693
Jameson, Dr. J. T., on Inversion of the Uterus, ....................... 716

L. PAGE.

Laryngitis, Acute, by Prof. T. F. Rochester, ....................... 18
La Roche on Yellow Fever, ......... 345
Lectures in Reply to Prof. West, .... 61
Lexicon, Reese's Medical, ........... 236
" Pronouncing, ............. 446
Libel Suit, ................... 447, 702
Lungs, Glucogenetic Function of, ... 370
Lunacy, Criminal, .................. 563

M.

Malaria, ........................... 126
" and Pneumonia, by Floyd Morse, M. D., ..................... 266
Mackenzie on the Eye, ............. 360
Maxson, Dr. E. R., on Exophthalmia, 585
" on Typhoid Fever, ........ 713
Medical Journals, .................. 616
" Jurisprudence, Wharton and Stillé's, ......................... 605
" Services, Value of, ........... 332
" Literature, .................. 633
" Gossip, ..................... 567
" Advertising, ................ 187
" College, Buffalo, . 188, 252, 318, 638
" and Surg. Association, Buffalo, . 757
Meacham, Dr. William G., on Local Treatment of Diseases of Air Passages, ............................ 69
" on Scarlatina, ............... 212
Medicine in Turkey, ................ 743
Mercurial Stomatitis, ............... 556
Meteorology, Practical, Sketch of, by Prof. S. B. Hunt, ................ 76
" Army, Register of, ........... 603
" of Buffalo, 35, 96, 164, 237, 315, 366, 411, 479, 543, 609, 656, 726
Michigan, University of, ....... 636, 755
Military Surgery, .................. 165
Mineral Waters, ................... 728
Morse, M. D., Floyd, on Pneumonia and Malaria, ...................... 266

N.

Nelaton's Clinical Surgery, ......... 478
Newman, Dr. James M., on Amer. Medical Association, .............. 587
Nourishment in Fevers, ............. 176

### O.

PAGE.

Obituary, ........................ 251
" of M. Valleix, ............ 318
" of Dr. Rufus W. Root, .... 703
" of Andrew Crosse, ....... 318
" of Moreton Stillé, ......... 319
" of Dr. Henry Clark, ....... 759
Obstetrics, Simpson's, .............. 469
Obstruction of Intestines, ........... 546

### P.

Pathological Society, ............... 239
" Anatomy, Rokitansky's, 358
Palmer, Case of Walter, ............ 687
Paralysis, Todd on, ................ 354
Parturition, Arrested by Calculus, .. 554
" Compression of Soft Parts during, ........................ 553
Pancreas, Pathology of, ............ 490
Paronychia, Analysis of Cases of, by Austin Flint, Jr., ............... 257
Pharmacopœia, Practitioner's, ....... 608
Phthisis, ........................ 109
Placenta Prævia, by Dr. D. W. Hershey, ........................ 141
Placenta Prævia, .................. 551
Pneumonia, Treatment of, .......... 38
" " " .......... 113
" Again, ................ 189
" and Malaria, by Floyd Morse, M. D., ................. 266
Pneumonia, by Wm. Van Pelt, M.D., 340
" Report on, by Prof. Flint, ..... 289
" Letter on, from Prof. Flint, ... 513
" Report on, by C. C. F. Gay, M.D. 271
" by Geo. N. Burwell, M. D., 87, 142 275, 417, 705
Polypus of the Uterus, ............ 126
Porcelain Teeth, ................. 253
Premature Labor, Induction of, ..... 97
Presentation of Post-mortem Instruments to Dr. Isodore Gluck, ....... 756
Professional Etiquette, ............ 243
Prostitution and Syphilis, .......... 675

### Q.

Quarantine, ...................... 185

### R.

Rand's Medical Chemistry, ......... 33

PAGE

Reception of Amer. Med. Association, 128
Reduplication of Both Sounds of the Heart, by Prof. Austin Flint, ..... 1
Records of the Soffolk District Med. Society, Extracts from, .......... 740
Retraction, ....................... 319
Registration of Births, ............. 507
Rochester, Prof. Thos. F., on Acute Laryngitis, ..................... 18
Rokitansky's Path. Anatomy, ....... 358
Robinson, Dr. L. G., on the Vitality of the Blood, ................... 385
R., on Biographies of Living Medical Men, ......................... 460
Root, M. D., John, Record of Deaths in Buffalo, 36, 94, 162, 238, 316, 964 412, 480, 544.
" Annual Report of, as Health Physician of the City of Buffalo, for 1855, ...................... 758

### S.

Salutatory, ....................... 60
Seat of War, ...................... 125
Scapula, Fractures of, by Prof. F. H. Hamilton, ..................... 449
Scarlatina, Contagiousness of, by Wm. C. Butler, M. D., ........... 534
" by Wm. G. Meacham, M. D., ... 212
Scarlatina, ....................... 486
Scenes in the Practice of a New York Surgeon, ...................... 382
Sciatica, ......................... 556
Simpson's Obstetrics, .............. 469
Small-pox and Vaccinia, ............ 102
Smelfungus, Letter from, ........... 445
Smith on Women, .................. 155
Skin, Lectures on Diseases of, by Prof. Austin Flint, .......... 129, 394
Socialistic Theories in their Medical Aspect, ........................ 374
" " " " 751
South American Physicians, ........ 561
Spiritualism, ..................... 500
Stokes on Heart and Aorta, ........ 360
Stone on Yellow Fever, ............ 549
Surgery, Nelaton's, ............... 478
Surgical Operations, .............. 619
Syphilis and Prostitution, .......... 675

PAGE.

Syringes, Glass, Female, by Prof. F. H. Hamilton, ..... 464

### T.

Tape Worm, ..... 105
Tanner's Manual, ..... 363
Todd on Paralysis, ..... 354
Transactions of Medical Society of Southern New York, ..... 363
" Amer. Med. Association, ..... 473
" Erie County Med. Society, 120, 524
Tribute to the Norfolk Martyrs, ..... 381
Tuberculosis, ..... 482, 692

### U.

Urinary Abscess, by C. B. Hutchins, M. D., ..... 577
" Organs, Gross on, ..... 160
" Calculi of Tennessee, ..... 62
Uterus, Extirpation of, ..... 40

PAGE.

Uterus, Polypus of, ..... 126
" Rupture of, ..... 497

### V.

Vaccinia and Small-pox, ..... 102
Varia, ..... 110
Vagina, Stricture of, by Wm. C. Butler, M. D., ..... 411
Van Pelt, Dr. Wm., on Pneumonia, ..... 340
" " on Epithelial Cancer, ..... 227

### W.

White, Prof. James P., Annual Address, by, ..... 529
" Case of Cyanosis, ..... 65
Women, Smith and Ashwell on, ..... 155

### Y.

Yellow Fever, Barton on, ..... 313
" " La Roche on, ..... 345

*Notice.*—To obviate a misapprehension which may arise from the retirement of the late senior editor of this journal, we are requested to state that Prof. Flint's residence will be at Buffalo until the session of lectures commences at Louisville, in autumn.

---

Our next number will contain a report of a case of cyanosis, with three fine lithographic illustrations, reported to the Buffalo Medical Association by Prof. James P. White.; also an article from the pen of Dr. Wm. G. Meacham, of Warsaw, N. Y., on the local treatment of diseases of the air passages.

